# Extensive Myocardial Calcification Following Fulminant Neonatal Enteroviral Myocarditis

**DOI:** 10.7759/cureus.97057

**Published:** 2025-11-17

**Authors:** Inês Noites, Sofia Guedes, Madalena Ferreira, Cristina Camilo, Francisco Abecasis

**Affiliations:** 1 Pediatric Intensive Care Unit, Pediatrics Department, Unidade Local de Saúde de Santa Maria, Lisbon, PRT; 2 Pediatrics Department, Hospital do Divino Espírito Santo de Ponta Delgada, Ponta Delgada, PRT; 3 Pediatrics Department, Unidade Local de Saúde da Lezíria, Hospital de Santarém, Santarém, PRT; 4 Pediatrics Department, Hospital de Cascais Dr. José de Almeida, Cascais, PRT

**Keywords:** cardiac failure, dystrophic myocardial calcification, enterovirus, neonatal myocarditis, va-ecmo

## Abstract

Myocardial calcification is a rare but important complication of fulminant neonatal myocarditis, a potentially life-threatening condition. Only a handful of neonatal cases have been reported in the literature. We present the case of a previously healthy seven-day-old term male neonate who developed cardiogenic shock secondary to enteroviral myocarditis with confirmed central nervous system involvement. Despite prompt initiation of inotropic therapy, he required venoarterial extracorporeal membrane oxygenation (VA-ECMO) within 48 hours of admission. While on mechanical circulatory support, serial imaging showed progressively increased myocardial echogenicity on echocardiography and later curvilinear myocardial calcification on chest radiograph. The clinical course was characterised by persistent cardiac dysfunction and progression to irreversible multiorgan failure. Heart transplantation was deemed unfeasible, and life-sustaining therapies were withdrawn, following multidisciplinary discussion and family consultation. An autopsy was not performed. This case highlights myocardial calcification as a potential imaging marker of irreversible myocardial injury in fulminant neonatal myocarditis. Early recognition of progressive myocardial echogenicity and subsequent calcification may provide valuable prognostic information, helping clinicians assess disease severity and support informed decision-making in cases of fulminant neonatal myocarditis.

## Introduction

Fulminant neonatal myocarditis is an uncommon but life-threatening inflammatory condition characterised by rapid onset of severe myocardial dysfunction and hemodynamic instability [1,2]. Viral pathogens, particularly enteroviruses such as Coxsackievirus B and Echovirus, are the most frequent etiologies in this age group [2,3].

Early diagnosis is challenging due to nonspecific clinical and laboratory findings, with echocardiography serving as the primary tool for cardiac function assessment, though it cannot confirm etiology [1]. Neonatal enterovirus infection presents with a broad clinical spectrum, from asymptomatic or mild symptoms to life-threatening conditions including myocarditis, meningoencephalitis, acute hepatitis, coagulopathy, and multiorgan failure [2].

In severe cases, extensive myocardial injury may lead to dystrophic calcification, a rare, ominous finding indicating irreversible myocardial damage [4]. The mechanism of dystrophic calcification is not completely understood. In fulminant myocarditis, the myocardium shows dystrophic calcification after necrosis, and myocardial necrosis is thought to result from myocardial inflammatory edema [5]. To the best of our knowledge, to date, fewer than five cases of this type of complication have been reported in the literature in the context of neonatal fulminant myocarditis, although it has been described in other age groups under similar circumstances. This complication has been associated with poor outcomes [1,4-6], especially in infants requiring extracorporeal life support. Mechanical circulatory support, including venoarterial extracorporeal membrane oxygenation (VA-ECMO), is often employed as a lifesaving measure and bridge to recovery or transplantation [4]. We report a fatal case of fulminant neonatal enteroviral myocarditis complicated by widespread myocardial calcification and refractory cardiac failure despite prolonged ECMO support.

## Case presentation

A previously healthy seven-day-old term male neonate presented to a local emergency department with poor feeding, anuria for over 12 hours, and progressive lethargy. He had been exclusively breastfed and exhibited poor weight gain since discharge. The mother reported a mild upper respiratory tract illness during the week preceding delivery. On examination, the newborn was afebrile but showed signs of circulatory collapse, including hypotension, bradycardia, mottled skin, delayed capillary refill, and cool extremities.

Initial laboratory evaluation (Table 1) revealed metabolic acidosis, severe thrombocytopenia, markedly elevated cardiac enzymes, and elevated inflammatory markers. Cerebrospinal fluid analysis revealed pleocytosis and elevated protein levels, raising concern for central nervous system involvement. Nasal polymerase chain reaction (PCR) for common respiratory viruses and blood and urine cultures were negative. 

**Table 1:** Main initial laboratory test results aPTT: activated partial thromboplastin time; CK: creatine kinase; CK-MB: creatine kinase-myocardial band; CMV: cytomegalovirus; CSF: cerebrospinal fluid; EBV: Epstein-Barr virus; HHV-6: human herpesvirus 6; HSV-1/2: herpes simplex virus type 1 and type 2; INR: international normalized ratio; NA: not applicable; NT-proBNP: N-terminal pro B-type natriuretic peptide; PCR: polymerase chain reaction; PT: prothrombin time; RSV: respiratory syncytial virus; SARS-CoV-2: severe acute respiratory syndrome coronavirus 2; VZV: varicella-zoster virus

Analytical parameters	Value	Reference interval
Gasimetry		
pH	7.13	7.35-7.45
Lactate (mmol/L)	4.88	0.50-1.80
Hematology		
Hemoglobin (g/dL)	18.1	12.5-20.5
Leukocytes (/μL)	22,410	6,000-22,000
Platelets (/μL)	16,000	160,000-500,000
Renal function		
Urea (mg/dL)	76	11-36
Creatinine (mg/dL)	0.35	<0.5
Liver enzymes		
Aspartate aminotransferase (U/L)	518	<40
Alanine aminotransferase (U/L)	99	<45
Alkaline phosphatase (U/L)	117	120-450
Gamma-glutamyl transferase (U/L)	225	0-60
Total bilirrubin (mg/dL)	8.48	0.2-1.0
Direct bilirrubin (mg/dL)	0.03	<0.20
Cardiac markers		
Troponin I (ng/L)	25,423	<58
NT-proBNP (ng/L)	>35,000	<300
CK (U/L)	1119	39-308
CK-MB (ng/mL)	34	<3.6
Inflammatory markers		
C-reactive protein (mg/dL)	1.65	<0.50
Procalcitonin (mg/dL)	3.90	<0.10
Coagulation		
PT (s)	18.7	11.8-15.1
INR	1.86	Varies according to condition
aPTT (s)	50.7	23-35
Fibrinogen (mg/dL)	117	169-515
CSF analysis		
White cell count (/μL)	60	<30
Protein (mg/dL)	138	20-120
Glucose (mg/dL)	63	60-80
Microbiology		
Nasal PCR	Negative for adenovirus, RSV, influenza A/B, SARS-CoV-2	NA
Blood culture	Negative	NA
Urine culture	Negative	NA
CSF PCR virus test	Negative for EBV, CMV, HSV-1/2, VZV, HHV-6	NA
CSF culture	Negative	NA

At 24 hours, echocardiography revealed severe left ventricular systolic dysfunction with global hypokinesis, nondilated ventricles, dilated atria, and moderate mitral and tricuspid regurgitation. Despite initiation of inotropic support, including noradrenaline, milrinone, dopamine, and adrenaline, he remained hemodynamically unstable, with a mean arterial pressure of 20-30 mmHg. 

Within 48 hours of presentation, the neonate required VA-ECMO via the right jugular vein and right carotid artery for refractory cardiogenic shock. He was subsequently transferred to a tertiary pediatric intensive care unit (PICU) for further management.

The infant initially received empirical broad-spectrum antibiotic therapy with ampicillin, cefotaxime, and gentamicin. Shortly after PICU admission, enterovirus was detected in bronchial secretions, cerebrospinal fluid, and stool samples, confirming the diagnosis of fulminant enteroviral myocarditis with associated meningoencephalitis. During VA-ECMO support, the infant developed several healthcare-associated infections, including methicillin-susceptible *Staphylococcus aureus* bacteremia, *Acinetobacter junii*, and *Stenotrophomonas maltophilia* respiratory infections, followed by *Pseudomonas aeruginosa* sepsis and *Candida parapsilosis* colonization. Each infection was managed with targeted antimicrobial therapy guided by culture results and susceptibility testing. Laboratory investigations performed during the PICU stay are summarised in Table 2.

**Table 2:** Main laboratory test results in the pediatric intensive care unit aPTT: activated partial thromboplastin time; CSF: cerebrospinal fluid; HHV-6: human herpesvirus 6; INR: international normalized ratio; NA: not applicable; NT-proBNP: N-terminal pro B-type natriuretic peptide; PCR: polymerase chain reaction; PT: prothrombin time; RSV: respiratory syncytial virus

Analytical parameters	Value	Reference interval
Hematology		
Hemoglobin (g/dL)	9.1	9.4-13.0
Leukocytes (/μL)	14,600	5,000-15,000
Platelets (/μL)	56,000	210,000-650,000
Renal function		
Urea (mg/dL)	51	16-49
Creatinine (mg/dL)	0.79	0.16-0.39
Liver enzymes		
Aspartate aminotransferase (U/L)	206	0-40
Alanine aminotransferase (U/L)	41	0-41
Lactate dehydrogenase (U/L)	2282	100-250
Alkaline phosphatase (U/L)	107	122-469
Gamma-glutamyl transferase (U/L)	111	0-60
Total bilirrubin (mg/dL)	24.9	<1.2
Direct bilirrubin (mg/dL)	15.07	<0.2
Cardiac markers		
Troponin I (ng/L)	52	<14
NT-proBNP (ng/L)	85,224	<450
Inflammatory markers		
C-reactive protein (mg/dL)	21.2	<0.5
Procalcitonin (mg/dL)	3.26	<0.5
Coagulation		
PT (s)	15.4	11.8-15.1
INR	1.34	Varies according to condition
aPTT (s)	68	23-35
Fibrinogen (mg/dL)	211	200-400
Microbiology		
Bronchial secretion PCR	Negative for RSV, rhinovirus, adenovirus, influenza A/B, parainfluenza 1–3, metapneumovirus; positive for enterovirus	NA
CSF PCR virus test	Negative for coxsackie, parvovirus B19, HHV-6, adenovirus; positive for enterovirus	NA
Stool PCR test	Positive for enterovirus	NA

**Figure 1:**
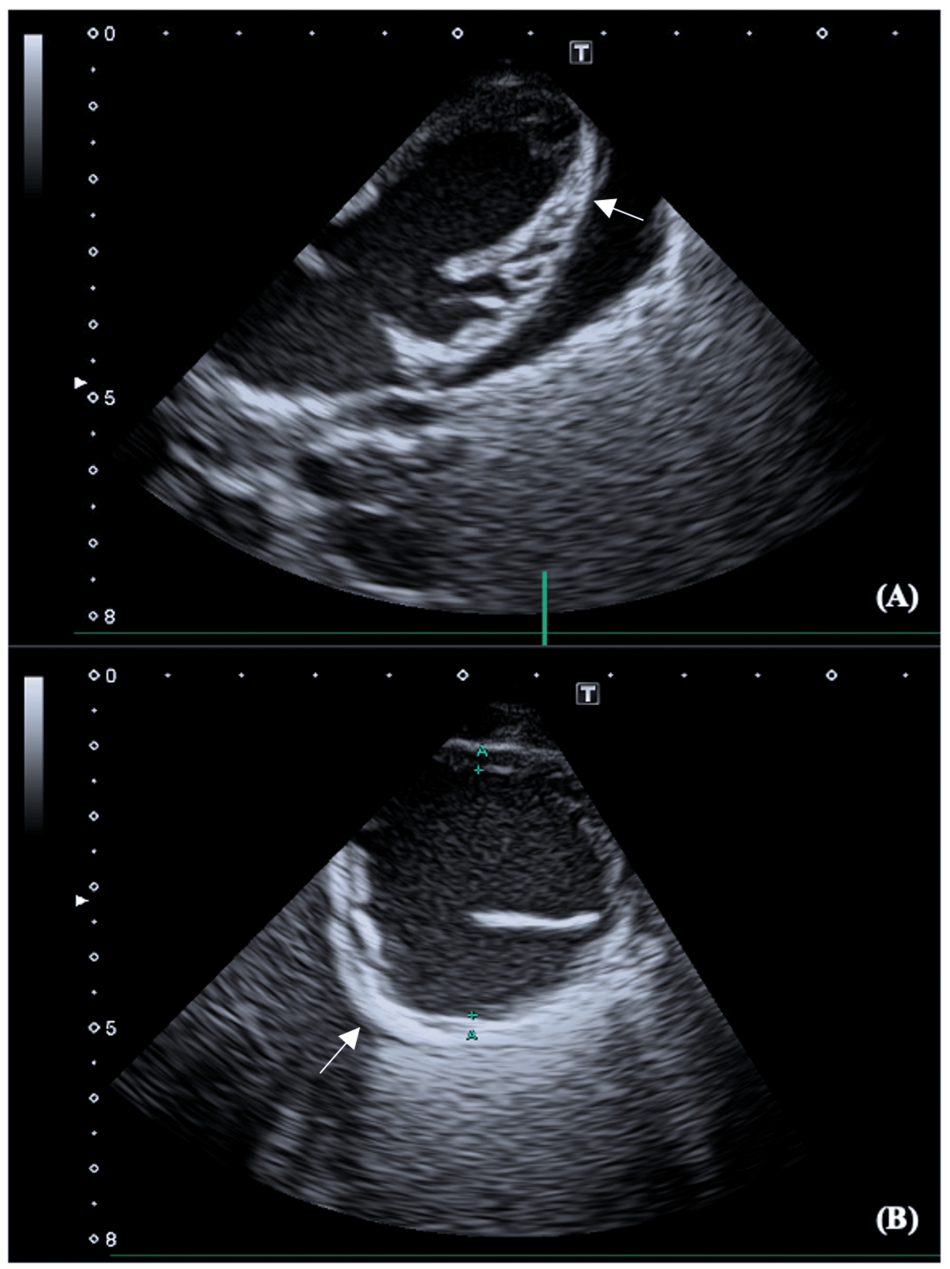
Two-dimensional echocardiogram at 50 days of life Subcostal window view (A) and parasternal short-axis view (B) showing diffuse hyperechoic areas (arrows) in the basal lateral wall. This finding is clinically significant as it may indicate evolving dystrophic calcification and irreversible myocardial injury

Over the following days, the neonate developed severe multiorgan dysfunction, including acute kidney injury requiring continuous hemodiafiltration, hepatic failure with progressive cholestasis, and worsening sepsis secondary to previously described nosocomial infections. Hematologic complications included persistent coagulopathy and thrombocytopenia requiring frequent transfusions. Despite ongoing ECMO support, serial echocardiograms revealed progressive dilation of the left heart chambers, persistent mitral regurgitation, and increasing myocardial echogenicity, most prominently in the basal lateral wall (Figure 1).

**Figure 2:**
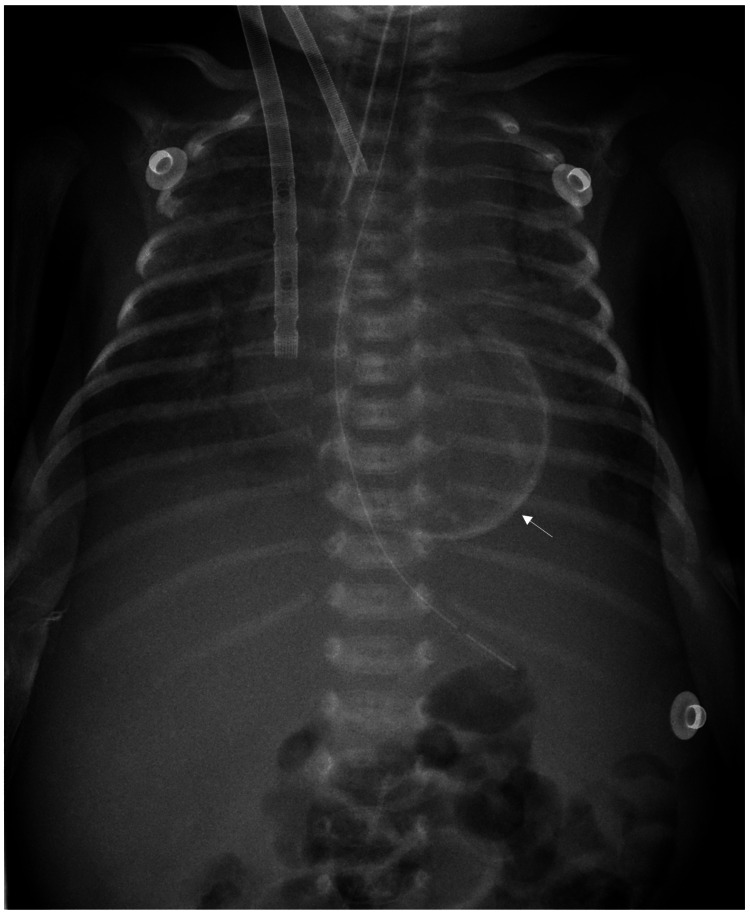
Chest radiograph showing myocardial calcification (arrow), visible as a smooth, eggshell-like curvilinear outline encircling the cardiac silhouette. The presence of this radiographic feature indicates advanced myocardial injury and correlates with poor prognosis

At 24 days of life, 17 days after admission, chest radiographs revealed abnormal curvilinear calcific outlines along the cardiac silhouette, suggestive of myocardial calcification (Figure 2).

Despite aggressive medical and supportive therapy, cardiac function remained severely impaired, with persistent closure of the aortic valve. Due to ongoing cardiac failure, he was listed for heart transplantation while on ECMO at 25 days of age. Nevertheless, his condition continued to deteriorate. By day 42 of ECMO support (at 50 days of age), a multidisciplinary team determined that heart transplantation was no longer feasible due to persistent multiorgan failure. Following consultation with the family and considering the poor prognosis and absence of further therapeutic options, a decision was made to withdraw life-sustaining measures. Sedation and analgesia were optimized to ensure comfort, and both ECMO and hemodiafiltration were discontinued at 53 days of life (45 days after initial admission). The patient died shortly thereafter. An autopsy was not performed.

## Discussion

Neonatal fulminant myocarditis is a rare but catastrophic condition, typically presenting with nonspecific symptoms and rapidly progressing to cardiogenic shock and multiorgan failure [1,2]. Viral infections, particularly enteroviruses, remain the predominant etiology in this age group [2,3]. This case illustrates the fulminant clinical course of enteroviral myocarditis, marked by rapid clinical deterioration, progressive multiorgan dysfunction, and ultimately extensive myocardial calcification and death, despite maximal medical management, including prolonged VA-ECMO support.

A particularly striking feature in this case was the development of dystrophic myocardial calcification within three weeks of disease onset, a rare complication reported only in a limited number of neonatal myocarditis cases [2,3,5]. Among the four previously reported neonates who required ECMO support in the setting of fulminant myocarditis with myocardial calcification, the calcification typically appeared during the second or third week of illness, and two of the four (50%) did not survive [1,4].

Echocardiography can detect increased myocardial echogenicity early in the disease course, potentially serving as a marker of evolving calcification and irreversible myocardial injury [1,3,5,6]. Early recognition of this finding may prompt closer monitoring, timely consideration of advanced circulatory support, or earlier evaluation for transplant candidacy. When progressive echogenicity occurs in conjunction with multiorgan dysfunction and a poor likelihood of cardiac recovery, early discussions regarding goals of care and palliative options may be warranted [7]. In our case, progressive myocardial echogenicity on serial echocardiography preceded radiographic evidence of calcification, underscoring the value of early and repeated imaging to assess myocardial integrity and guide prognostic evaluation. 

Myocardial calcification in neonatal myocarditis is strongly associated with poor outcomes, particularly in neonates requiring mechanical circulatory support [3,4]. Although radiography has a limited diagnostic role, it remains a simple and widely available imaging modality that can support the diagnosis of fulminant myocarditis with myocardial calcification [4]. Visible calcifications on radiographs generally indicate advanced myocardial injury [3,4] and carry important prognostic implications [4]. This finding aligns with our experience, underscoring myocardial calcification as a surrogate marker of irreversible myocardial damage and a potential predictor of nonrecovery despite aggressive therapeutic interventions.

The American College of Cardiology recommends endomyocardial biopsy in patients with advanced (stage D) heart failure, including those with severe cardiac dysfunction or mechanical circulatory support, when the diagnosis remains uncertain or when histopathologic findings could alter management [7]. The diagnostic yield is highest within the first two weeks of symptom onset; however, in the presence of dystrophic calcification, suggesting irreversible myocardial injury, the likelihood of identifying treatable active myocarditis is reduced. Furthermore, procedural risks are substantially increased during ECMO, including cardiac tamponade, myocardial perforation, sustained ventricular arrhythmias, and bleeding [8,9]. In this context, endomyocardial biopsy was not pursued, as the etiology was established and the risks outweighed the limited potential to influence prognosis. Antiviral or immunomodulatory therapies (e.g. intravenous immunoglobulin or corticosteroids) were not administered, reflecting ongoing uncertainty regarding their efficacy in fulminant neonatal enteroviral myocarditis [7]. Reports of antiviral use, such as pocapavir, remain limited and largely anecdotal, with insufficient evidence to suggest improved outcomes once severe myocardial injury has occurred [7]. Accordingly, the management strategy in this case focused on supportive care rather than experimental interventions with unproven benefit in this setting.

In this case, persistent multiorgan failure, combined with minimal likelihood of myocardial recovery and limited availability of neonatal donor hearts, ultimately rendered heart transplantation unfeasible. This observation aligns with prior reports indicating that ECMO may serve as a bridge to either recovery or transplantation, but its utility is limited when myocardial recovery is unlikely and transplantation is not an option [3,4].

Collectively, these factors highlight the critical importance of timely, comprehensive, and multidisciplinary evaluation of transplant candidacy in neonates with fulminant myocarditis. Prolonged ECMO support in the absence of cardiac recovery, particularly when accompanied by myocardial calcification and progressive multiorgan dysfunction, risks extending invasive interventions that may ultimately prove futile. Clinicians must carefully balance efforts to pursue recovery or transplantation with the need to initiate compassionate, family-centred palliative care when further curative treatment is no longer appropriate. A limitation of this report is the absence of post-mortem histopathologic confirmation of myocardial calcification. Although imaging findings strongly suggest extensive dystrophic calcification, an autopsy would have enabled more precise characterisation of its extent and distribution.

## Conclusions

Myocardial calcification in fulminant neonatal myocarditis represents an ominous prognostic marker, indicating irreversible myocardial injury. Early detection of evolving calcification and associated dysfunction through serial echocardiography and radiography is essential to prompt urgent, multidisciplinary evaluation and reassessment of treatment goals. Decisions regarding the withdrawal of life-sustaining therapies should be guided by a comprehensive clinical, ethical, and logistical framework, including transplant eligibility and donor availability. This case highlights the devastating nature of fulminant neonatal myocarditis complicated by myocardial calcification and suggests that such findings may carry important prognostic significance. Given its rarity, further multicentre studies or systematic reviews are needed to validate imaging-based prognostic markers and to develop consensus guidelines that support compassionate, timely, and ethically grounded decision-making in neonatal cardiac critical care.
